# Assessment of Chronic Postsurgical Pain After Knee Replacement: A Systematic Review

**DOI:** 10.1002/acr.22050

**Published:** 2013-11-01

**Authors:** Vikki Wylde, Julie Bruce, Andrew Beswick, Karen Elvers, Rachael Gooberman-Hill

**Affiliations:** 1 University of BristolBristol, UK; 2 University of WarwickCoventry, UK

## Abstract

**Objective**Approximately 20% of patients experience chronic pain after total knee replacement (TKR), yet there is no consensus about how best to assess such pain. This systematic review aimed to identify measures used to characterize chronic pain after TKR.

**Methods**MEDLINE, Embase, PsycINFO, Cochrane Library, and CINAHL databases were searched for research articles published in all languages from January 2002 to November 2011. Articles were eligible for inclusion if they assessed knee pain at a minimum of 3 months after TKR, yielding a total of 1,164 articles. The data extracted included the study design, country, timings of assessments, and outcome measures containing pain items. The outcome measures were compared with domains recommended by the Initiative on Methods, Measurement, and Pain Assessment in Clinical Trials (IMMPACT) for inclusion in the assessment of chronic pain–related outcomes within clinical trials. Temporal trends were also explored.

**Results**The review found use of a wide variety of composite and single-item measures, with the American Knee Society Score the most common. Many measures used in published studies did not capture the multidimensional nature of pain recommended by the IMMPACT; of those commonly used, the Western Ontario and McMaster Universities Osteoarthritis Index and Oxford Knee Score were the most comprehensive. Geographic trends were evident, with nation-specific preferences for particular measures. A recent reduction in the use of some clinically administered tools was accompanied by an increased use of patient-reported outcome measures.

**Conclusion**There was wide variation in the methods of pain assessment alongside nation-specific preferences and changing temporal trends in pain assessment after TKR. Standardization and improvements in assessment are needed to enhance the quality of research and facilitate the establishment of a core outcome set.

## INTRODUCTION

Total knee replacement (TKR) is one of the most common elective surgical procedures, with 81,979 operations performed in the NHS during 2010 (1). The procedure is performed to provide pain relief and restore physical functioning; it can also improve health-related quality of life and enable some people to return to social, leisure, and sporting activities (2,3). Although TKR is successful in providing pain relief for the majority of patients, ∼20% of patients continue to experience chronic pain in their replaced knee (5). The potential burden of this pain is considerable, particularly given recent evidence suggesting a trebling in rates of TKR surgery within the UK over a 16-year period (6).

Chronic postsurgical pain (CPSP) is defined as pain that occurs after a surgical procedure and lasts for at least 2 months (7). However, the International Association for the Study of Pain recognizes that the timeframe in the definition of CPSP may vary according to surgery type (8). Pain severity generally plateaus at 3 months after TKR (9) and therefore for the purposes of this research, chronic pain after TKR was defined as pain that is present 3 months after surgery.

The expectation of pain relief is a primary reason why patients elect to undergo TKR (10), and it is crucial that research assesses whether this expectation has been met. For clinical trials investigating efficacy of chronic pain treatments, the Initiative on Methods, Measurement, and Pain Assessment in Clinical Trials (IMMPACT) recommends that outcome assessment should include domains that reflect the multidimensional nature of pain (pain, physical functioning, emotional functioning, participant ratings of global improvement, symptoms and adverse events, and participant disposition 11). According to the IMMPACT guidelines, the assessment of pain should encompass pain severity, pain medication usage, pain quality, and the temporal aspects of pain.

Currently, there is no agreement among studies in the literature on which measures should be used to assess chronic pain after TKR. One recent systematic review identified outcome measures used in 53 randomized controlled trials (RCTs) of TKR and found extensive variation in the tools that were employed (12). However, this review included only trials published in English over a 7-year period and focused on functional outcome measures rather than pain specifically. The use of many different outcome measures renders comparisons across studies and meta-analyses problematic (13). An agreement on core outcomes would allow comparative research and facilitate the development of multicenter clinical databanks or repositories.

Given the burden and distress related to chronic pain after TKR (14), there is a clear need to identify and consider measures that provide an appropriate assessment of pain. The purpose of this study was to undertake a comprehensive and systematic review of contemporary studies to identify measures used to assess chronic pain after TKR. Our review aimed to determine which pain instruments and broader health-related measures incorporating pain items were used in original epidemiologic and experimental research articles published over a 10-year period. Within these articles, we explored geographic and contemporaneous trends in the use of pain-related data collection instruments.

**Significance & Innovations**

- This systematic review identifies that there is wide variation in the methods used for pain assessment after knee replacement.
- This systematic review highlights the need to improve consistency and quality in the assessment and reporting of chronic pain after total knee replacement.

## MATERIALS AND METHODS

### Literature search

We used systematic review methods in accordance with the Meta-Analysis of Observational Studies in Epidemiology proposal for reporting systematic reviews and meta-analyses of observational studies (15) (see Supplementary Appendix A, available in the online version of this article at http://onlinelibrary.wiley.com/doi/10.1002/acr.22050/abstract). MEDLINE, Embase, PsycINFO, Cochrane Library, and CINAHL databases were searched for articles published from January 1, 2002 to November 22, 2011. The study design filters were not applied to the search strategies in order to maximize the number of hits obtained. The search strategies were modified for different bibliographic databases. No language restrictions were applied. The search terms included combinations of terms, such as “arthroplasty, replacement, prosthesis, implant, knee, pain, outcomes.” Truncation terms and synonyms were used to maximize the efficiency of the search. Full details of the search terms can be found in Supplementary Appendix B (available in the online version of this article at http://onlinelibrary.wiley.com/doi/10.1002/acr.22050/abstract).

### Inclusion and exclusion criteria

Published original research articles reporting data from all study designs were eligible for inclusion if they assessed pain at a minimum of 3 months after primary TKR. Studies were excluded if they were case studies (or if they recruited fewer than 10 participants), conference abstracts, PhD theses, reviews, editorials, or any publication that did not present original primary data. Studies that assessed a mixed cohort of patients (e.g., knee and hip replacement patients) were included in the review and only data relevant to the TKR patients were extracted.

### Eligibility screening

All articles identified in the search were imported and stored in EndNote X5 (Thomson Reuters). Abstracts or full-text articles were screened by a member of the research team (VW) to determine if they met the eligibility criteria. The reasons for excluding articles were recorded as free text in EndNote X5.

### Data extraction

A standardized data extraction form was used to extract data from eligible articles and data were entered into a Microsoft Access database. The data extracted included the study objective, study design, setting, country of the first author, number of study participants recruited, timings of assessments, and outcome measures that contained pain items. All screening and data extraction were performed by 1 reviewer with postgraduate qualifications in the field of pain after joint replacement (VW). Because of the large volume of articles that were identified in the literature search, it was impractical to perform duplicate screening and data extraction on all studies; however, we acknowledged that single data extraction is associated with the probability of an increased number of errors (16). Therefore, blind duplicate screening was performed (AB) on a 10% subsample of the references retrieved (n = 851), which found that the primary reviewer missed 1 eligible article, suggesting that few eligible studies may have been missed. Thereafter, blind duplicate data extraction was performed (KE) on a 5% subsample of full-text articles (n = 63); full agreement was found between the primary and secondary reviewers on the type and content of the outcome measures that assessed pain.

Where data could not be extracted from the full-text article because the article was unobtainable, data were extracted from the abstract. Attempts were made to contact the authors of these studies to verify the accuracy of the extracted data. Data were extracted from 184 abstracts and the research team successfully traced contact details for 84 authors. These authors were e-mailed and asked to complete a validation form or return a copy of the full-text article. Replies were received from 36 authors (14 authors returned a completed validation form and 22 authors provided full-text copies of their article).

### Statistical analysis

Descriptive statistics were used to show the number of different outcome measures that assessed pain. The outcome measures used were then categorized into 2 types: multi-item tools were measures that included ≥1 questions about pain among other questions (e.g., the Oxford Knee Score [OKS]) and single-item questions consisted of only 1 question about pain (e.g., visual analog scale [VAS] for pain). For all multi-item and single-item pain-related questions, details of the question content and response options were extracted and coded to explore which aspects of pain were captured. Preliminary coding was performed by 1 researcher (VW) and all codes were checked independently by a second researcher (AB, JB, or RG-H). Development of the coding framework was based on the IMMPACT guidelines for pain assessment in chronic pain trials (11). Thus, the questions were coded into domains that can be assessed by outcome measures (pain, physical functioning, emotional functioning, and participant ratings of global improvement). The pain domain was further broken down into pain severity, use of pain medications, pain quality, and temporal aspects of pain.

## RESULTS

### Characteristics of the included studies

**Figure 1:**
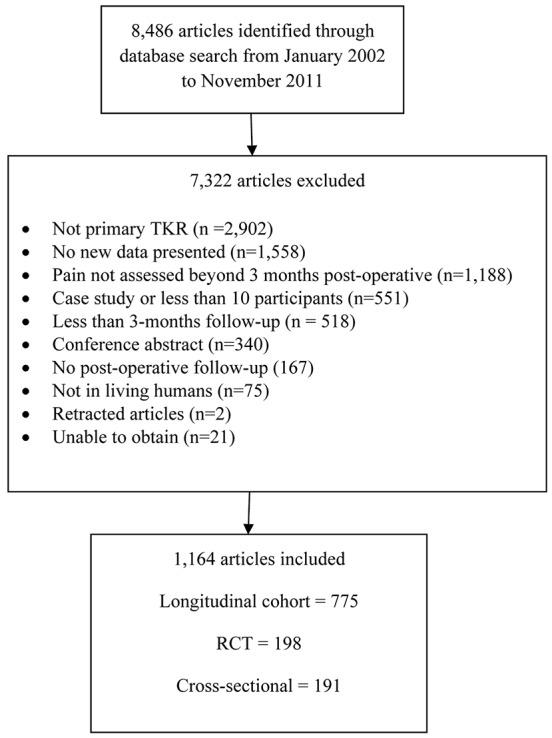
Systematic review flow chart. TKR = total knee replacement; RCT = randomized controlled trial.

A total of 8,486 articles were identified in the study search and screened for eligibility, with 1,164 (13.7%) meeting the selection criteria. The reasons for excluding articles are shown in Figure 1. Of the 1,164 included studies, 775 were cohort studies, 198 were RCTs, and 191 were cross-sectional studies. The duration of postoperative followup ranged from 3 months to 18 years. The number of TKR patients recruited could be determined for 1,149 studies (99%), with a total of 316,247 patients recruited (range 10–13,627 patients). In the 1,164 articles, the outcome measures that assessed pain were used 1,990 times. The studies used a variable number of outcome measures that incorporated pain items (range 1–14), with 658 studies (57%) using 1 measure, 304 (26%) using 2 measures, 138 (12%) using 3 measures, and 64 (5%) using ≥4 measures. Multi-item tools containing pain questions were used 1,657 times, and single-item pain tools were used 333 times.

### Multi-item tools

Overall, 54 different multi-item tools containing pain questions were used in the TKR studies. The 5 most commonly used multi-item tools were the American Knee Society Score (AKSS) (17), Western Ontario and McMaster Universities Osteoarthritis Index (WOMAC) (18), Hospital for Special Surgery Knee Score (HSS) (19), Short Form 36 (SF-36) (20), and OKS (21). The details of the multi-item tools that were used in >5 studies and the number of items that assessed pain within each of these tools are shown in Table 1. Eleven of the 18 multi-item tools included only a single question about pain.

**Table 1:** Multi-item tools used in >5 studies*

Name of multi-item tool	No. of studies that used tool (%)	No. of items in tool	No. of items in tool assessing pain
American Knee Society Score	675 (58)	10	1
WOMAC	267 (23)	24	5
Hospital for Special Surgery Knee Score	184 (16)	7	2
Short Form 36	165 (14)	36	2
Oxford Knee Score	101 (9)	12	5
Short Form 12	54 (5)	12	1
Knee Injury and Osteoarthritis Outcome Score	26 (2)	42	9
EQ-5D	25 (2)	5	1
Feller Patellar Score	20 (2)	4	1
Knee Outcome Survey activities of daily living scale	14 (1)	17	1
Lequesne Index	11 (<1)	12	5
Tegner and Lysholm Score	9 (<1)	8	1
Total Knee Function Questionnaire	9 (<1)	55	1
Nottingham Health Profile	7 (<1)	45	8
Self-Administered Patient Satisfaction Scale	6 (<1)	4	1
Stern and Insall Patellar Score	6 (<1)	1	1
Bristol Knee Score	6 (<1)	9	1
15D	6 (<1)	15	1

* WOMAC = Western Ontario and McMaster Universities Osteoarthritis Index; EQ-5D = EuroQol 5-domain instrument; 15D = 15-dimensional instrument.

Table2 shows an overview of the aspects of pain assessed by each of the 5 most commonly used multi-item tools, classified according to the IMMPACT recommendations for pain assessment (11). Pain severity and pain during physical functioning were the only dimensions assessed by all of the multi-item tools; none of the tools assessed pain quality, pain medication use, or participant ratings of global improvement. In terms of the number of questions included that assessed pain and the breadth of pain dimensions captured, the OKS and WOMAC provided the most comprehensive assessments of chronic pain.

**Table 2:** Aspects of pain assessed by the 5 most commonly used multi-item tools*

	AKSS	WOMAC	HSS	SF-36	OKS
No. of pain questions in tool	1	5	2	2	5
Pain severity	1 question (pain severity)	5 questions (pain severity on different activities)	2 questions (pain severity on rest and walking)	1 question (bodily pain severity)	2 questions (average pain severity and pain severity standing from chair)
Use of pain medications	–	–	–	–	–
Pain quality (affective and sensory qualities of pain)	–	–	–	–	–
Temporal aspects of pain (e.g., variability, pattern, frequency, and duration)	1 question (response options of occasional or continual pain)	1 question (pain severity at night)	–	–	1 question (frequency of being troubled by pain at night)
Physical functioning (pain-related interference with activity or movement)	1 question (response options of pain severity on walking and climbing stairs)	4 questions (pain severity on walking, climbing stairs, sitting or lying, and standing upright)	1 question (pain severity on walking)	1 question (interference of pain with normal work)	3 questions (interference of pain with normal work, distance walked before pain becomes severe, and severity of pain on standing from chair)
Emotional functioning (pain-related emotional distress)	–	–	–	–	2 questions (being troubled by pain at night and response option of unbearable pain)
Participant ratings of global improvement	–	–	–	–	–

* AKSS = American Knee Society Score; WOMAC = Western Ontario and McMaster Universities Osteoarthritis Index; HSS = Hospital for Special Surgery Knee Score; SF-36 = Short Form 36; OKS = Oxford Knee Score.

A breakdown of the most commonly used health-related quality of life, disease-specific, and joint-specific tools is shown in Supplementary Appendix C (available in the online version of this article at http://onlinelibrary.wiley.com/doi/10.1002/acr.22050/abstract). The AKSS was the most commonly used joint-specific tool, the SF-36 was the most commonly used health-related quality of life tool, and the WOMAC was the most commonly used disease-specific tool.

#### Geographic trends in the use of multi-item tools

**Figure 2:**
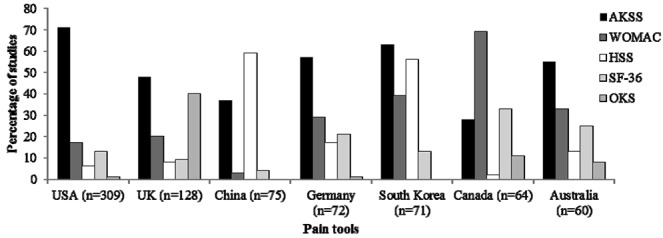
Geographic trends in the use of multi-item tools. AKSS = American Knee Society Score; WOMAC = Western Ontario and McMaster Universities Osteoarthritis Index; HSS = Hospital for Special Surgery Knee Score; SF-36 = Short Form 36; OKS = Oxford Knee Score.

The use of multi-item tools by countries that contributed >50 articles to the review (the US, the UK, China, Germany, South Korea, Canada, and Australia) was compared (Figure 2). Together, these countries published 779 (67%) of the studies included in this review. The percentage of studies from each country that used the 5 most common multi-item tools was compared. Nation-specific preferences for particular tools were apparent, with the AKSS being the most commonly used tool in studies published from the US, the UK, Germany, South Korea, and Australia. The HSS was most commonly used in studies from China, whereas the WOMAC was the tool most frequently used in Canadian studies. Over the past 10 years, the OKS was predominantly used in studies from the UK.

#### Multi-item tools by study design

A comparison of the 5 most commonly used multi-item tools by study design is shown in Supplementary Appendix D (available in the online version of this article at http://onlinelibrary.wiley.com/doi/10.1002/acr.22050/abstract). The use of these multi-item tools was reasonably similar across RCTs, cohort studies, and cross-sectional studies.

#### Temporal trends in the use of multi-item tools

**Figure 3:**
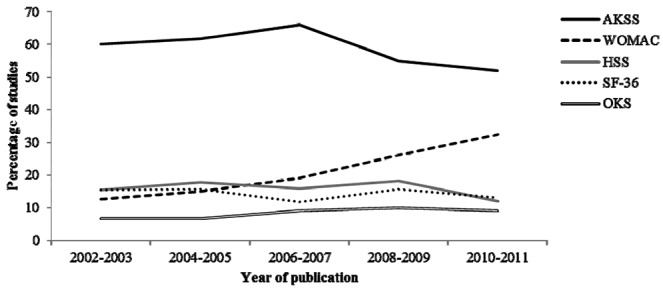
Temporal trends in the use of multi-item tools. AKSS = American Knee Society Score; WOMAC = Western Ontario and McMaster Universities Osteoarthritis Index; HSS = Hospital for Special Surgery Knee Score; SF-36 = Short Form 36; OKS = Oxford Knee Score.

The percentage of studies using the 5 most commonly used multi-item tools over a 10-year period is shown in Figure 3. Over the past 5 years, there has been a reduction in the proportion of studies that have used the AKSS (from 66% of studies in 2006–2007 to 52% of studies in 2010–2011). Over the same time period, there has been an increase in the proportion of studies that have used the WOMAC (from 19% of studies in 2006–2007 to 32% of studies in 2010–2011).

### Single-item questions

Single-item questions were used 333 times in 228 studies to assess chronic pain after TKR. The aspects of pain assessed by the single-item questions on the basis of the framework provided by the IMMPACT are shown in Table3. Pain severity was the most frequently assessed aspect of pain, with 68% of single-item questions providing a measurement of pain severity. The pain VAS was the most commonly used question format to assess pain severity. Only a small percentage of the single-item tools assessed pain medication use, pain quality, temporal aspects of pain, emotional functioning, or participant ratings of global improvement.

**Table 3:** Aspects of pain assessed by the single-item questions*

Pain domain	Examples of codes	No. of single-item questions (%)
Pain severity	Average pain severity, worst pain severity, and presence/absence of pain	227 (68)
Use of pain medications	Frequency of use, adherence, and decreased need	8 (2)
Pain quality	Location of pain (e.g., anterior knee pain)	57 (17)
Temporal aspects of pain	Pain frequency, night pain, and intermittent pain	33 (10)
Physical functioning	Pain on walking, pain on climbing stairs, pain during sports, and pain at rest	98 (29)
Emotional functioning	Unbearable pain, bothersome pain, and emotional well-being	5 (1.5)
Participant ratings of global improvement	Satisfaction with pain relief, reduction in pain from operation, and fulfillment of expectations	26 (8)

* Each single-item pain question could be coded into >1 pain domain.

## DISCUSSION

This is the first systematic review to assess the methods used for the measurement of chronic pain in epidemiologic and experimental studies of TKR. This review involved mapping the existing literature, exploring trends in the use of outcome measures to assess chronic pain, and comparing the assessment of chronic pain after TKR with recent guidance from the IMMPACT. Previous systematic reviews have found variation in the assessment of generic outcomes in musculoskeletal and orthopedic clinical trials (12–22). This review found extensive variation in the outcome measures used to assess chronic pain after TKR, adding to the existing knowledge base through a focus on the assessment of CPSP. Of the 5 most common multi-item tools used, the OKS and WOMAC provided the most comprehensive assessment of chronic pain, although they did not assess all of the pain-related domains that the IMMPACT recommends should be measured in clinical trials investigating the efficacy of chronic pain treatments. Future studies investigating the outcome after TKR could incorporate either the OKS or WOMAC to capture basic features of pain; however, other multidimensional measures would provide a more comprehensive assessment of chronic pain.

There are numerous tools available to assess general health and functional outcomes after TKR (12), but there is no agreement on which tool provides the optimal assessment of pain. A key finding of this review was that, despite a growing interest in investigating the burden, characteristics, and impact of chronic pain after TKR, the assessment of pain has been inconsistent. The gaps in pain assessment involve 3 main areas. First, many studies reporting outcomes after TKR failed to include any assessment of pain; a total of 1,188 articles were excluded from the review because they did not include any assessment of pain beyond 3 months postsurgery. In light of the current evidence about the prevalence of chronic pain after TKR (5), this is highly problematic. Second, the most commonly used multi-item tools include only a small number of questions about pain; this is because the primary focus of many of these tools is the assessment of function or general health. Although readily available, few studies have used established pain assessment tools such as the McGill Pain Questionnaire (23) or the Chronic Pain Grade (24) to capture the intensity, characteristics, and limitations associated with chronic pain after TKR. These and other pain assessment tools provide comprehensive mapping of pain characteristics, and omission of these tools from studies means that chronic pain after TKR remains poorly quantified and characterized. Third, the pain tools used were predominantly oriented toward assessing pain severity and there was little assessment of other key aspects of pain, such as temporality and quality, as recommended by the IMMPACT (11). While we acknowledge that 222 (19%) of the articles included in this review predate the release of the IMMPACT guidelines in 2005 and that a similar number published in 2006–2007 may have been designed prior to dissemination of the guidelines, the majority of studies published after 2007 did not assess the pain domains recommended by the IMMPACT. Although the IMMPACT guidelines were developed to provide guidance for the assessment of chronic pain within clinical trials, a comparison of the IMMPACT recommendations with wider clinical and epidemiologic TKR literature highlights that commonly used measurement methods fail to capture the broader postoperative pain experience. Along with understanding the causes of pain, a comprehensive assessment of pain is central to the development and evaluation of interventions and improvements to clinical practice. Most TKR procedures are conducted as a way of managing osteoarthritis (OA), and recent developments in OA pain assessment may signal the start of interest in more comprehensive assessments of pain before and after TKR. For example, the assessment of qualities of OA pain may assist in the identification of pain of a neuropathic nature (25), which may prove useful in informing future assessments of chronic pain after TKR. In addition, more comprehensive OA pain measures have been developed and are starting to be used (e.g., the measure of Intermittent and Constant Osteoarthritis Pain) (26).

This review found that the AKSS was overwhelmingly the most commonly used outcome measure to assess chronic pain after TKR. This is true for other interventions, with reviews finding that the AKSS has been one of the tools most commonly reported upon in orthopedic studies (12–27). The AKSS includes only a single question on pain with multiple response options; the scale involves a clinician-conducted assessment and calculation of a composite score based on pain, functional ability, and measurements such as range of motion and joint stability. Although it is widely used, the AKSS was not formally validated during its development and subsequent studies assessing its psychometric properties have identified limitations such as a low correlation between items and poor inter- and intraobserver reliability (28–29). Furthermore, clinician-administered tools have been widely criticized because of the recognized discordance between the views of patients and clinicians (30–31). It is therefore apparent that, despite its extensive use, the AKSS has a limited utility in the assessment of pain-related postoperative outcome. This suggests that continued use of the AKSS represents a conservative approach to outcome assessment in orthopedics, with convention hindering progression. However, our review identified a slight reduction in the use of the AKSS over time accompanied by an increased use of the WOMAC, which may herald a change due to an increased awareness of the importance of assessing outcome from the perspective of the patient. In the UK, this change is reflected in the national patient-reported outcome measures (PROMs) initiative, which collects Oxford Hip and Knee Scores on all patients undergoing elective primary lower extremity replacement in the NHS (32). In the US and other countries, PROMs are increasingly promoted as an appropriate way to collect information about patient outcomes (33–34). Future research will be required to explore if this early trend away from clinician-administered tools and toward PROMs continues in orthopedics. Additionally, future research could explore the trends in data collection methods (e.g., postal or online questionnaire versus data collected during clinic appointments) and the cost implications of these different methods.

International variation in the use of multi-item tools was found, with some of the more popular tools used more frequently in some countries than others. These trends for nation-specific preferences for particular tools to assess TKR outcomes are similar to those of studies found in the general orthopedic literature (35). For example, the US published the highest percentage of studies using the AKSS, while the UK published the highest percentage of studies using the OKS. The greater use of tools in the countries that developed them is not unexpected, although it could pose difficulties for international comparisons of outcomes in meta-analyses (13). The finding of international variation in the use of tools suggests that standardization of pain assessment on an international level may prove difficult; however, an alternative could be the promotion of national standardization through the merging of large cohort data sets to create registries or data repositories. This is one of the aims of the Core Outcome Measures in Effectiveness Trials initiative, which promotes the standardization of outcomes assessment through the establishment of core outcome sets (36).

It is important to acknowledge the limitations of this review when interpreting the results. First, although contact with authors yielded some full-text articles and data extraction validation, extraction of data from abstracts alone represented 13% of the data set. This may have led to an underestimation of the number of tools used to assess pain because data may not have been reported in the abstract. Second, it is also possible that we underestimated the number of studies and tools used to assess pain; some studies may have assessed but not reported pain data (reporting bias). Third, because of the high volume of studies, it was not feasible to analyze the assessment of pain according to study quality.

The strengths of this review were the wide inclusion criteria that enabled scrutiny of a high volume of original epidemiologic and experimental research articles, systematic and rigorous methods used to search and screen eligible articles, and attempts to contact authors. The inclusion of literature published over a 10-year period in all languages enabled comparisons of temporal and geographic trends in the use of pain-related outcome measures. This systematic review highlights that the assessment of chronic pain after TKR could be improved. Future research is needed to develop a consensus and standardization on which pain domains should be assessed after TKR; this can be achieved by working to establish a core outcome set and subsequent guidance on the most suitable outcome measures for assessment of the core pain domains after TKR.

## AUTHOR CONTRIBUTIONS

All authors were involved in drafting the article or revising it critically for important intellectual content, and all authors approved the final version to be published. Dr. Wylde had full access to all of the data in the study and takes responsibility for the integrity of the data and the accuracy of the data analysis.

**Study conception and design.** Wylde, Bruce, Beswick, Gooberman-Hill.

**Acquisition of data.** Wylde, Bruce, Beswick, Elvers.

**Analysis and interpretation of data.** Wylde, Bruce, Beswick, Gooberman-Hill.
