# Supplementary material for: Assessment of Chronic Postsurgical Pain After Knee Replacement: A Systematic Review
Source: Arthritis Care Res (Hoboken). 2013 Nov 1;65(11):1795–803. doi: 10.1002/acr.22050 (PMC3883092; doi:10.1002/acr.22050)
Supplement: Supplementary file 1 [file acr0065-1795-sd1.doc]

**Supplementary material 1: MOOSE checklist for systematic reviews**

| **Guidelines** | **How these guidelines were addressed in the systematic review** |
| --- | --- |
| Reporting of background should include: |  |
| Problem definition | Introduction. How is chronic pain after total knee replacement assessed? |
| Hypothesis statement | Introduction. To determine which tools are used to assess chronic pain after total knee replacement |
| Description of study outcome | Introduction/methods. Tools used to assess pain at a minimum of 3-months after total knee replacement |
| Type of exposure or intervention used | Introduction. Total knee replacement |
| Type of study designs used | Methods. All study designs except case studies and studies which recruited less than 10 patients |
| Study population | Methods. Patients who had undergone total knee replacement with a minimum post-operative follow-up of 3 months |
| Reporting of search strategy should include |  |
| Qualifications of searchers | Methods. Researchers experienced in systematic reviews, pain assessment and orthopaedics |
| Search strategy, including time period included in the synthesis and keywords | Methods and Appendix 1. Databases search from 1^st^ January 2002 to 22^nd^ November 2011. Search terms provided in Appendix 1 |
| Effort to include all available studies, including contact with authors | Methods. Eighty four authors were contacted to verify information extracted from abstracts |
| Databases and registries searched | Methods. Databases searched included MEDLINE, Embase, PsycINFO, Cochrane Library and CINAHL databases |
| Search software used, name and version, including special features used | Methods. No search software was used. |
| Use of hand searching | Not performed because of extensive volume of literature identified in the literature search |
| List of citations located and those excluded, including justification | Details provided in Figure 1 |
| Method of addressing articles published in languages other than English | Methods. Data was extracted from the abstract, and then authors whose contact details could be traced were contacted to verify extracted data |
| Method of handling abstracts and unpublished studies | Methods. Conference proceedings and unpublished studies were excluded |
| Description of any contact with authors | Methods. Eighty four authors were contacted to verify information extracted from abstracts |
| Reporting of methods should include: |  |
| Description of relevance or appropriateness of studies assembled for assessing the hypothesis to be tested | Methods/results. All research (excluding case studies and studies with less than 10 participants) published over a 10 year period which assessed pain at a minimum of 3-months after total knee replacement were included |
| Rationale for the selection and coding of data | Methods/results. Data on the tools used to assess chronic pain after total knee replacement was extracted |
| Documentation of how data were classified and coded | Methods/results. Eligibility screening performed in duplicate by two researchers for 851 (10% sample) articles and data extraction performed in duplication for 63 (5% sample) articles. |
| Assessment of confounding | Not performed |
| Assessment of study quality | Not performed |
| Assessment of heterogeneity | Not performed |
| Description of statistical methods | Methods. Meta-analysis not performed, descriptive statistics used to present data |
| Provision of appropriate tables and graphics | Methods. Data summarised in four tables and four figures |
| Reporting of results should include: |  |
| Graphic summarizing individual study estimates and overall estimate | Not relevant – data analysis was focussed on identifying which tools are used to assess chronic pain after total knee replacement |
| Table giving descriptive information for each study included | Not included due to high volume of literature included in review |
| Results of sensitivity testing | Not performed |
| Indication of statistical uncertainty of findings | Not relevant |
| Reporting of discussion should include: |  |
| Quantitative assessment of bias | Discussion. It is acknowledged that pain may have been assessed in some studies but not reported. |
| Justification for exclusion | No exclusions on the basis of language of publication |
| Assessment of quality of included studies | Discussion. This was not performed and is acknowledged as a limitation in the discussion |
| Reporting of conclusions should include |  |
| Consideration of alternative explanations for observed results | Discussion. It is acknowledged that pain may have been assessed in some studies but not reported. |
| Generalization of the conclusions | Discussion. It is acknowledged that the results are only applicable to the assessment of pain after one type of operation and in the time frame specified |
| Guidelines for future research | Discussion. The review identified some temporal trends in the use of tools to assess chronic pain after total knee replacement, which will require further investigation in the future |
| Disclosure of funding source | Funding described |
