# Supplementary material for: Assessment of Chronic Postsurgical Pain After Knee Replacement: A Systematic Review
Source: Arthritis Care Res (Hoboken). 2013 Nov 1;65(11):1795–803. doi: 10.1002/acr.22050 (PMC3883092; doi:10.1002/acr.22050)
Supplement: Supplementary file 2 [file acr0065-1795-sd2.doc]

**Supplementary material 2: SEARCH TERMS**

**MEDLINE search terms**

1. exp Arthroplasty, Replacement, Knee/

2. knee prosthesis.mp. or exp Knee Prosthesis/

3. exp Arthroplasty, Replacement, Knee/ or exp Knee Prosthesis/ or knee implant.mp.

4. exp Arthroplasty, Replacement, Knee/ or exp Knee Prosthesis/ or knee replacement.mp.

5. (knee$ adj5 (arthroplast$ or replacement$ or implant$ or prothes$)).mp.

6. 1 or 2 or 3 or 4 or 5

7. exp Pain/ or exp Complex Regional Pain Syndromes/ or exp Pain Clinics/ or exp Pain, Postoperative/ or exp Pain, Intractable/ or exp Pain Measurement/ or exp Patellofemoral Pain Syndrome/ or exp Pain, Referred/ or pain.mp. or exp Pain Threshold/ or exp Pain Perception/

8. (pain adj5 (chronic or persistent or long-term)).mp.

9. analgesi$.mp.

10. ache$.mp.

11. discomfort$.mp.

12. outcome$.mp.

13. neuropath$.mp.

14. 7 or 8 or 9 or 10 or 11 or 12 or 13

15. 6 and 14

**EMBASE search terms**

1. knee replacement.mp. or exp knee arthroplasty/

2. knee prosthesis.mp. or exp knee prosthesis/

3. knee arthroplasty.mp. or exp knee arthroplasty/

4. exp total knee replacement/ or exp knee prosthesis/ or knee implant.mp. or exp knee arthroplasty/

5. (knee$ adj5 (arthroplast$ or replacement$ or implant$ or prothes$)).mp.

6. 1 or 2 or 3 or 4 or 5

7. exp complex regional pain syndrome type II/ or pain assessment/ or exp referred pain/ or exp pain parameters/ or exp neuropathic pain/ or exp pain threshold/ or exp complex regional pain syndrome/ or exp patellofemoral pain syndrome/ or exp musculoskeletal pain/ or exp pain/ or exp limb pain/ or exp knee pain/ or exp leg pain/ or pain.mp. or exp McGill Pain Questionnaire/ or exp complex regional pain syndrome type I/ or exp postoperative pain/ or exp intractable pain/ or exp Brief Pain Inventory/ or exp pain clinic/

8. (pain adj5 (chronic or persistent or long-term)).mp.

9. analgesi$.mp.

10. ache$.mp.

11. discomfort$.mp.

12. outcome$.mp.

13. neuropath$.mp.

14. 7 or 8 or 9 or 10 or 11 or 12 or 13

15. 6 and 14

**PsychINFO search terms**

1. knee replacement.mp.

2. knee arthroplasty.mp.

3. knee prosthesis.mp.

4. knee implant.mp.

5. (knee$ adj5 (arthroplast$ or replacement$ or implant$ or prothes$)).mp.

6. 1 or 2 or 3 or 4 or 5

7. exp Pain Measurement/ or exp Neuropathic Pain/ or exp Pain Perception/ or exp Somatoform Pain Disorder/ or exp Pain Thresholds/ or exp Pain/ or exp Chronic Pain/ or pain.mp. or exp Pain Management/

8. (pain adj5 (chronic or persistent or long-term)).mp.

9. analgesi$.mp.

10. ache$.mp.

11. discomfort$.mp.

12. outcome$.mp.

13. neuropath$.mp.

14. 7 or 8 or 9 or 10 or 11 or 12 or 13

15. 6 and 14

**Cochrane search terms**

#1 knee near/5 replacement

#2 MeSH descriptor Arthroplasty, Replacement, Knee explode all trees

#3 knee near/5 arthroplasty

#4 knee near/5 implant

#5 knee near/5 prosthesis

#6 (#1 OR #2 OR #3 OR #4 OR #5)

#7 pain

#8 MeSH descriptor Pain explode all trees

#9 analgesi*

#10 ache*

#11 discomfort*

#12 outcome*

#13 neuropath*

#14 (#7 OR #8 OR #9 OR #10 OR #11 OR #12 OR #13)

#15 (#6 AND #14)

**Cinahl search terms**

S13 S5 and S12 Search modes

S12 S6 or S7 or S8 or S9 or S10 or S11 Search modes

S11 neuropath*

S10 outcome*

S9 discomfort*

S8 ache*
S7 analgesi*
S6 pain Search modes
S5 S1 or S2 or S3 or S4
S4 knee implant Expanders - Apply related words
S3 knee prosthesis Expanders - Apply related words

S2 knee arthroplasty Expanders - Apply related words

S1 knee replacement Expanders - Apply related words
