# Supplementary material for: Assessment of Chronic Postsurgical Pain After Knee Replacement: A Systematic Review
Source: Arthritis Care Res (Hoboken). 2013 Nov 1;65(11):1795–803. doi: 10.1002/acr.22050 (PMC3883092; doi:10.1002/acr.22050)
Supplement: Supplementary file 3 [file acr0065-1795-sd3.doc]

**Supplementary material 3**: Five most commonly used joint-specific, disease-specific and health-related quality of life tools

| **Name of multi-item tool** | **Number of studies (%) that used tool** |
| --- | --- |
| **Joint-specific tools** |  |
| American Knee Society Score | 675 (58%) |
| Hospital for Special Surgery Knee Score | 184 (16%) |
| Oxford Knee Score | 101 (9%) |
| Knee injury and Osteoarthritis Outcome Score | 26 (2%) |
| Feller Patellar Score | 20 (2%) |
| **Disease-specific tools** |  |
| WOMAC | 267 (23%) |
| Self-Administered Patient Satisfaction Scale | 6 (<1%) |
| Enneking Score | 5 (<1%) |
| AIMS-2 | 3 (<1%) |
| Self-Efficacy for Rehabilitation Outcome Scale | 3 (<1%) |
| **Health-related quality of life tools** |  |
| Short Form-36 | 165 (14%) |
| Short Form-12 | 54 (5%) |
| EQ-5D | 25 (2%) |
| Nottingham Health Profile | 7 (<1%) |
| 15D | 6 (<1%) |
