# Supplementary figures and images for: Assessment of Chronic Postsurgical Pain After Knee Replacement: A Systematic Review
Source: Arthritis Care Res (Hoboken). 2013 Nov 1;65(11):1795–803. doi: 10.1002/acr.22050 (PMC3883092; doi:10.1002/acr.22050)

**
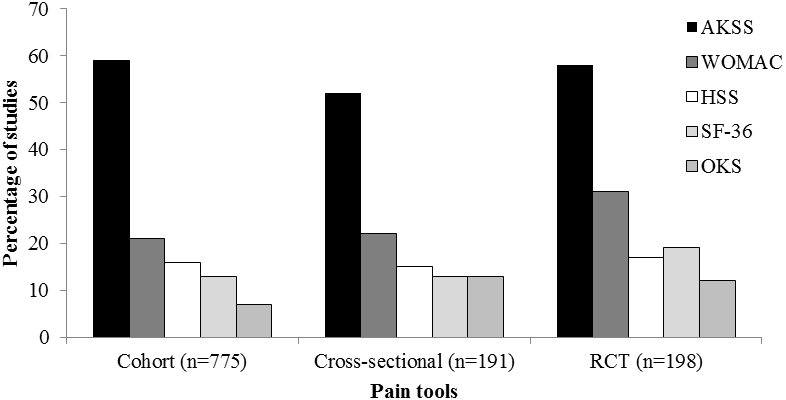
**

**Supplementary material 4: Use of multi-item tools by study design**

Supplement: Supplementary file 4 [file acr0065-1795-sd4.doc]
